# Supplementary material for: A Personalized, Symptom‐Based Approach to Boost Research Participation in Hospitalized Older Adults
Source: J Am Geriatr Soc. 2026 Aug 4;74(9):2688–96. doi: 10.1111/jgs.70622 (PMC13589679; doi:10.1111/jgs.70622)
Supplement: Supplementary file 1 [file JGS-74-2688-s001.pdf]

Supplementary Material

A Personalized, Symptom-Based Approach to Boost Research Participation in Hospitalized Older Adults

Nicole Ceriani, BA[1], Shreya Dhar, BA[1], Catherine Zhao, BA[1], Ian Sherrington, BA[1], Eyal Y. Kimchi, MD, PhD[1]

[1]Department of Neurology, Northwestern University Feinberg School of Medicine, Chicago, IL, USA

Supplementary Figure S 1 EEG wearable device

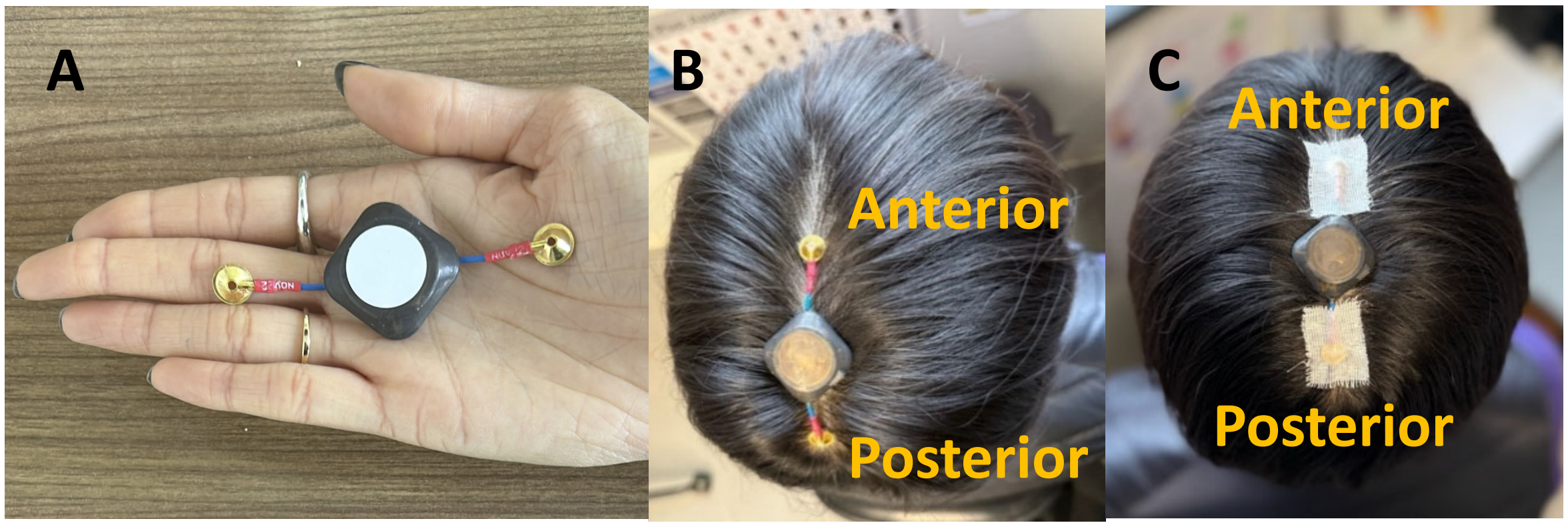

A The EEG device is approximately 25mm along each edge with gold cups attached at diagonal corners for placement at the vertex of the head.

B The device is shown at the vertex of the head with the anterior electrode at standard International EEG electrode location Cz, and with the posterior electrode 7cm behind at Pz.

C The device is affixed to the head using standard EEG application procedures including collodion and cotton gauze.

Supplementary Table S 1 Comorbidities and admission diagnoses of patients who enrolled in the study before and after interventions.

	Pre (n=68)	Post (n=58)	P-Value
**Charlson Comorbidities, n (%)**			
AIDS HIV	0 (0.0)	0 (0.0)	1.000
Cerebrovascular disease	17 (25.0)	18 (31.0)	0.579
Chronic pulmonary disease	16 (23.5)	21 (36.2)	0.173
Congestive heart failure	14 (20.6)	18 (31.0)	0.255
Dementia	4 (5.9)	4 (6.9)	1.000
Diabetes with chronic complications	7 (10.3)	6 (10.3)	1.000
Diabetes without chronic complications	11 (16.2)	12 (20.7)	0.673
Hemiplegia or paraplegia	1 (1.5)	1 (1.7)	1.000
Liver disease	5 (7.4)	7 (12.1)	0.552
Malignancy	11 (16.2)	14 (24.1)	0.372
Metastatic Solid Tumor	4 (5.9)	3 (5.2)	1.000
Myocardial infarction	2 (2.9)	0 (0.0)	0.497
Peptic ulcer disease	3 (4.4)	2 (3.4)	1.000
Peripheral vascular disease	17 (25.0)	20 (34.5)	0.333
Renal disease	13 (19.1)	12 (20.7)	1.000
Rheumatic disease	6 (8.8)	7 (12.1)	0.762
**Admission WHO ICD-10 Diagnosis Chapters, n (%)**			
Certain infectious and parasitic diseases	1 (1.5)	3 (5.2)	0.294
Codes for special purposes	0 (0.0)	1 (1.7)	
Diseases of the blood and blood-forming organs and certain disorders involving the immune mechanism	2 (2.9)	1 (1.7)	
Diseases of the circulatory system	11 (16.2)	9 (15.5)	
Diseases of the digestive system	1 (1.5)	1 (1.7)	
Diseases of the eye and adnexa	1 (1.5)	0 (0.0)	
Diseases of the genitourinary system	3 (4.4)	5 (8.6)	
Diseases of the musculoskeletal system and connective tissue	5 (7.4)	1 (1.7)	
Diseases of the nervous system	15 (22.1)	4 (6.9)	
Diseases of the respiratory system	3 (4.4)	6 (10.3)	
Endocrine, nutritional and metabolic diseases	2 (2.9)	5 (8.6)	
Factors influencing health status and contact with health services	2 (2.9)	2 (3.4)	
Mental and behavioural disorders	2 (2.9)	3 (5.2)	
Neoplasms	4 (5.9)	2 (3.4)	
Symptoms, signs and abnormal clinical and laboratory findings, not elsewhere classified	16 (23.5)	15 (25.9)	

Charlson comorbidities were extracted from the electronic medical record and compared using Chi-squared tests or Fisher tests when expected or actual cell counts were less than 5. P-values are not corrected for multiple comparisons given the exploratory and descriptive nature of these data. Admission diagnoses were extracted from the electronic medical record and classified according to World Health Organization (WHO) International Classification of Disease-10 (ICD-10) diagnosis chapters. A Fisher test was used to compare the distribution of diagnoses between the two groups.

Supplementary Table S 2 Comparison of demographics of enrolled patients to all potentially eligible patients admitted during the study period.

		Enrolled	All Admits	P-Value
**Patients n**		126	7702	
**Age, median [25th percentile, 75th percentile]**		72.0 [62.0,80.0]	62.0 [47.0,72.0]	<0.001
**Gender, n (%)**	**Female**	64 (50.8)	3694 (48.0)	0.357
	**Male**	61 (48.4)	3978 (51.6)	
	**Non-binary**	1 (0.8)	30 (0.4)	
**Race, n (%)**	**American Indian or Alaska Native**	0 (0.0)	17 (0.2)	0.056
	**Asian**	6 (4.8)	271 (3.5)	
	**Black or African American**	26 (20.6)	1419 (18.4)	
	**More than One Race**	2 (1.6)	39 (0.5)	
	**Native Hawaiian or Other Pacific Islander**	0 (0.0)	11 (0.1)	
	**Not Reported**	7 (5.6)	1054 (13.7)	
	**White**	85 (67.5)	4891 (63.5)	
**Ethnicity, n (%)**	**Hispanic or Latino**	12 (9.5)	569 (7.4)	0.464
	**Not Hispanic or Latino**	107 (84.9)	6536 (84.9)	
	**Not Reported**	7 (5.6)	597 (7.8)	
**Hospital Service, n (%)**	**General Medicine**	81 (64.3)	2795 (36.3)	<0.001
	**Hematology/Oncology**	7 (5.6)	1439 (18.7)	
	**Neurology**	31 (24.6)	876 (11.4)	
	**Neurosurgery**	4 (3.2)	348 (4.5)	
	**Surgery**	3 (2.4)	2244 (29.1)	
**Baseline Cognitive Impairment, n (%)**	**Absent**	111 (88.1)	7451 (96.7)	<0.001
	**Present**	15 (11.9)	251 (3.3)	

Quantitative and categorical data were compared between patients who enrolled vs. all eligible patients admitted during the study period. Age was compared using the Kruskal-Wallis test. Enrollment data and categorical variables were compared using Chi-squared tests or Fisher tests when cell counts were less than 5.

Supplementary Table S 3 Comorbidities and admission diagnoses of enrolled patients to all potentially eligible patients admitted during the study period.

	Enrolled (n=126)	All Admits (n=7702)	P-Value
**Charlson Comorbidities, n (%)**			
AIDS HIV	0 (0.0)	57 (0.7)	1.000
Cerebrovascular disease	35 (27.8)	1161 (15.1)	<0.001
Chronic pulmonary disease	37 (29.4)	1528 (19.8)	0.011
Congestive heart failure	32 (25.4)	1733 (22.5)	0.507
Dementia	8 (6.3)	95 (1.2)	<0.001
Diabetes with chronic complications	13 (10.3)	711 (9.2)	0.793
Diabetes without chronic complications	23 (18.3)	1529 (19.9)	0.739
Hemiplegia or paraplegia	2 (1.6)	181 (2.4)	1.000
Liver disease	12 (9.5)	1167 (15.2)	0.104
Malignancy	25 (19.8)	2464 (32.0)	0.005
Metastatic Solid Tumor	7 (5.6)	878 (11.4)	0.056
Myocardial infarction	2 (1.6)	328 (4.3)	0.209
Peptic ulcer disease	5 (4.0)	206 (2.7)	0.389
Peripheral vascular disease	37 (29.4)	1549 (20.1)	0.014
Renal disease	25 (19.8)	1762 (22.9)	0.485
Rheumatic disease	13 (10.3)	388 (5.0)	0.014
**Admission WHO ICD-10 Diagnosis Chapters, n (%)**			
Certain infectious and parasitic diseases	4 (3.2)	60 (0.8)	<0.001
Codes for special purposes	1 (0.8)	6 (0.1)	
Congenital malformations, deformations and chromosomal abnormalities	0 (0.0)	24 (0.3)	
Diseases of the blood and blood-forming organs and certain disorders involving the immune mechanism	3 (2.4)	143 (1.9)	
Diseases of the circulatory system	20 (15.9)	1287 (16.7)	
Diseases of the digestive system	2 (1.6)	568 (7.4)	
Diseases of the ear and mastoid process	0 (0.0)	10 (0.1)	
Diseases of the eye and adnexa	1 (0.8)	9 (0.1)	
Diseases of the genitourinary system	8 (6.3)	250 (3.2)	
Diseases of the musculoskeletal system and connective tissue	6 (4.8)	203 (2.6)	
Diseases of the nervous system	19 (15.1)	472 (6.1)	
Diseases of the respiratory system	9 (7.1)	206 (2.7)	
Diseases of the skin and subcutaneous tissue	0 (0.0)	65 (0.8)	
Endocrine, nutritional and metabolic diseases	7 (5.6)	190 (2.5)	
External causes of morbidity and mortality	0 (0.0)	16 (0.2)	
Factors influencing health status and contact with health services	4 (3.2)	1458 (18.9)	
Injury, poisoning and certain other consequences of external causes	0 (0.0)	341 (4.4)	

Mental and behavioural disorders	5 (4.0)	78 (1.0)	
Neoplasms	6 (4.8)	945 (12.3)	
Other	0 (0.0)	506 (6.6)	

Charlson comorbidities were extracted from the electronic medical record and compared using Chi-squared tests or Fisher tests when expected or actual cell counts were less than 5. P-values are not corrected for multiple comparisons given the exploratory and descriptive nature of these data. Admission diagnoses were extracted from the electronic medical record and classified according to World Health Organization (WHO) International Classification of Disease-10 (ICD-10) diagnosis chapters. A Fisher test was used to compare the distribution of diagnoses between the two groups.
